# A randomized clinical trial on the effects of exercise on muscle remodelling following bariatric surgery

**DOI:** 10.1002/jcsm.12815

**Published:** 2021-10-19

**Authors:** Saulo Gil, John P. Kirwan, Igor H. Murai, Wagner S. Dantas, Carlos Alberto Abujabra Merege‐Filho, Sujoy Ghosh, Samuel K. Shinjo, Rosa M.R. Pereira, Walcy R. Teodoro, Sheylla M. Felau, Fabiana B. Benatti, Ana L. de Sá‐Pinto, Fernanda Lima, Roberto de Cleva, Marco Aurélio Santo, Bruno Gualano, Hamilton Roschel

**Affiliations:** ^1^ Applied Physiology & Nutrition Research Group, School of Physical Education and Sport, Rheumatology Division, Faculdade de Medicina FMUSP Universidade de São Paulo São Paulo SP Brazil; ^2^ Laboratory of Assessment and Conditioning in Rheumatology Universidade de São Paulo São Paulo SP Brazil; ^3^ Rheumatology Division, Hospital das Clínicas HCFMUSP, Faculdade de Medicina Universidade de São Paulo São Paulo Brazil; ^4^ Integrated Physiology and Molecular Medicine Laboratory, Pennington Biomedical Research Center Louisiana State University Baton Rouge LA USA; ^5^ Laboratory of Computational Biology, Pennington Biomedical Research Center Louisiana State University Baton Rouge LA USA; ^6^ Centre for Computational Biology Duke‐NUS Medical School Singapore; ^7^ School of Applied Sciences Universidade Estadual de Campinas São Paulo Brazil; ^8^ Gastroenterology Department, Digestive Surgery Division Department of Digestive Division, Hospital das Clínicas HCFMUSP, Faculdade de Medicina Universidade de São Paulo São Paulo Brazil

**Keywords:** Muscle atrophy, Obesity, Bariatric surgery, Muscle function

## Abstract

**Background:**

Muscle atrophy and strength loss are common adverse outcomes following bariatric surgery. This randomized, controlled trial investigated the effects of exercise training on bariatric surgery‐induced loss of muscle mass and function. Additionally, we investigated the effects of the intervention on molecular and histological mediators of muscle remodelling.

**Methods:**

Eighty women with obesity were randomly assigned to a Roux‐en‐Y gastric bypass (RYGB: *n* = 40, age = 42 ± 8 years) or RYGB plus exercise training group (RYGB + ET: *n* = 40, age = 38 ± 7 years). Clinical and laboratory parameters were assessed at baseline, and 3 (POST3) and 9 months (POST9) after surgery. The 6 month, three‐times‐a‐week, exercise intervention (resistance plus aerobic exercise) was initiated 3 months post‐surgery (for RYGB + ET). A healthy, lean, age‐matched control group was recruited to provide reference values for selected variables.

**Results:**

Surgery resulted in a similar (*P* = 0.66) reduction in lower‐limb muscle strength in RYGB and RYGB+ET (−26% vs. −31%), which was rescued to baseline values in RYGB + ET (*P* = 0.21 vs. baseline) but not in RYGB (*P* < 0.01 vs. baseline). Patients in RYGB+ET had greater absolute (214 vs. 120 kg, *P* < 0.01) and relative (2.4 vs. 1.4 kg/body mass, *P* < 0.01) muscle strength compared with RYGB alone at POST9. Exercise resulted in better performance in timed‐up‐and‐go (6.3 vs. 7.1 s, *P* = 0.05) and timed‐stand‐test (18 vs. 14 repetitions, *P* < 0.01) compared with RYGB. Fat‐free mass was lower (POST9‐PRE) after RYBG than RYGB + ET (total: −7.9 vs. −4.9 kg, *P* < 0.01; lower‐limb: −3.8 vs. −2.7 kg, *P* = 0.02). Surgery reduced Types I (~ − 21%; *P* = 0.99 between‐group comparison) and II fibre cross‐sectional areas (~ − 27%; *P* = 0.88 between‐group comparison), which were rescued to baseline values in RYGB+ET (*P* > 0.05 vs. baseline) but not RYGB (*P* > 0.01 vs. baseline). RYGB + ET showed greater Type I (5187 vs. 3898 μm^2^, *P* < 0.01) and Type II (5165 vs. 3565 μm^2^, *P* < 0.01) fCSA than RYGB at POST9. RYGB + ET also resulted in increased capillarization (*P* < 0.01) and satellite cell content (*P* < 0.01) than RYGB at POST9. Gene‐set normalized enrichment scores for the muscle transcriptome revealed that the *ubiquitin‐mediated proteolysis* pathway was suppressed in RYGB + ET at POST9 vs. PRE (NES: −1.7; *P* < 0.01), but not in RYGB. *Atrogin‐1* gene expression was lower in RYGB + ET vs. RYGB at POST9 (0.18 vs. 0.71‐fold change, *P* < 0.01). From both genotypic and phenotypic perspectives, the muscle of exercised patients resembled that of healthy lean individuals.

**Conclusions:**

This study provides compelling evidence—from gene to function—that strongly supports the incorporation of exercise into the recovery algorithm for bariatric patients so as to counteract the post‐surgical loss of muscle mass and function.

## Introduction

Bariatric surgery is the treatment of choice for severe obesity as it yields substantial weight loss and reversal of cardiometabolic risk factors^1^; however, this procedure is not free of adverse outcomes. Some post‐operative patients exhibit significant loss of fat‐free mass,^2^ strength, and functionality,^3^, ^4^ which is important because these parameters are related to distinct physiological processes (e.g. immune response, regulation of glucose levels, protein synthesis, and basal metabolic rate),^5^, ^6^, ^7^ frailty syndrome,^8^ and most critically, mortality.^9^, ^10^

The underlying mechanisms related to muscle atrophy following bariatric surgery remain largely underexplored. Individuals with obesity are thought to exhibit a blunted anabolic response to stimuli like exercise bouts, a condition known as anabolic resistance.^11^ This resistance has been ascribed to (i) reduced activation of proteins related to the *mTOR* pathway (a master regulator of muscle protein synthesis)^12^; (ii) altered proteolytic activity (e.g. the ubiquitin‐proteasome system and autophagic/lysosomal system)^13^; (iii) microvascular rarefaction^14^; and (iv) reduced myogenic response.^15^ Whether and how these mechanisms contribute to surgery‐induced muscle atrophy remains to be determined.

Exercise improves muscle mass, strength, and functionality in a wide spectrum of sub‐populations; however, whether exercise preserves muscle mass in post‐bariatric patients is controversial.^16^, ^17^, ^18^, ^19^ Indeed, Stegen *et al*.^18^ observed higher muscle strength levels in post‐bariatric surgery patients undergoing 12 weeks of exercise training than non‐exercised patients; however, exercise did not affect fat‐free mass sparing or functionality. Conversely, Herring *et al*.^17^ demonstrated that 12 weeks of exercise training attenuated the loss in fat‐free mass and resulted in greater strength and functionality gains in patients undergoing bariatric surgery vs. a non‐exercised group. It is noteworthy that both studies evaluated fat‐free mass using electrical bioimpedance, a method that is neither an accurate measurement of fat‐free mass in individuals with obesity^20^ nor of muscle mass. To the best of our knowledge, the only study that directly assessed muscle mass (i.e. magnetic resonance imaging) in post‐bariatric surgery patients undergoing exercise training did not detect any effect of exercise on muscle mass sparing.^19^ The dissonant findings are hard to reconcile, and in fact, literature is scarce on this subject, warranting further investigation. Moreover, the studies investigating the effects of exercise on muscle mass and function of post‐bariatric surgery patients have failed to identify mechanisms underlying exercise‐mediated muscle remodelling in this population. We^21^, ^22^ and others^23^, ^24^ have shown that exercise promotes beneficial physiological and metabolic effects that are additive to the weight loss effects of surgery, including effects on bone mass, inflammatory markers, endothelial function, and insulin sensitivity.

Herein, we performed a randomized, controlled, mechanistic trial to comprehensively examine the effects of exercise training on body composition (fat‐free mass as primary outcome), muscle function and related cellular and molecular mechanisms (secondary outcomes) in women undergoing bariatric surgery. We hypothesized that exercise training following bariatric surgery would attenuate loss of fat‐free mass and conserve muscle function when compared with standard of care. We further proposed that histological and molecular analysis would generate new insights on the muscle remodelling mechanisms resulting from the surgical procedure and exercise.

## Methods

### Study design and human subjects

This was a randomized controlled trial (registered at Clinicaltrials.gov as NCT02441361) that *a priori* aimed to investigate the effects of exercise training following bariatric surgery on multiple clinical outcomes. Specifically, this manuscript focuses on the effects of exercise training on muscle mass and function after bariatric surgery, as well as in the molecular and histological mediators of muscle remodelling. Additional reports from this trial contain data on endothelial function, bone mass, and insulin resistance were published elsewhere.^21^, ^22^, ^25^ Data were collected between March 2015 and September 2019 in São Paulo (Brazil). The study was approved by the Ethics Committee of the Clinical Hospital of the School of Medicine of the University of São Paulo, and all procedures were in accordance with the recommendations of the Helsinki Declaration. All patients provided written informed consent before being enrolled in the study. This manuscript is reported according to the guidelines of the Consolidated Standards of Reporting Trials.

Patients were recruited from the Metabolic and Bariatric Surgery Unit of the Clinical Hospital of the School of Medicine of the University of São Paulo. Inclusion criteria were as follows: women who were eligible for bariatric surgery [body mass index (BMI) > 40 kg/m^2^ or ≥35 kg/m^2^ with associated comorbidities], 18–60 years, and not engaged in an exercise training programme for at least 1 year prior to the study. Exclusion criteria involved cancer in the past 5 years, and any cardiovascular diseases, neurological disorders, or skeletal muscle impairment that would contraindicate exercise practice. None of the patients were receiving hormone replacement therapy.

Before surgery, patients were randomly assigned (1:1) into either a Roux‐en‐Y gastric bypass (RYGB) or Roux‐en‐Y gastric bypass plus exercise training group (RYGB + ET), using a computer‐generated randomization code. Clinical and laboratory parameters were assessed at baseline (PRE), and 3 (POST3) and 9 (POST9) months after the surgery. The 6 month exercise intervention (for the exercise group) started at POST3 upon post‐surgery medical clearance for high‐intensity exercise participation. A healthy control group comprising lean, age‐matched women was also recruited and assessed to provide reference values for selected variables.

### Exercise intervention

The RYGB + ET group participated in a 6 month, three‐times‐a‐week, supervised exercise training programme at the hospital, whereas the RYGB group received standard post‐surgery care during follow‐up. Training sessions included a 5 min light warm‐up followed by strengthening exercises for the major muscle groups (leg‐press 45°, leg extension, half‐squat, bench press, lat pulldown, seated row, and calf raise) and aerobic exercise on a treadmill. The resistance exercise protocol was comprised of three sets of 8–12 repetition maximum with a 60 s rest interval between sets and exercises. Load progression (5%) was employed as soon as patients were able to perform two or more repetitions than previously determined. Aerobic training consisted of 30–60 min (10 min progression every 4 weeks) of treadmill walking at an intensity corresponding to 50% of the delta difference between the ventilatory anaerobic threshold and respiratory compensation point. Heart rate was monitored throughout every session to ensure proper exercise intensity (Polar®). Any adverse events or signs and symptoms were documented including feelings of general fatigue, soft tissue soreness, and injury or illness; and a record of attendance was kept ensuring adherence to the protocol.

### Body weight and composition

Body weight was assessed on a calibrated digital scale and height was evaluated with the aid of a stadiometer, from which BMI was calculated. All patients underwent a whole‐body dual‐energy x‐ray absorptiometry scan (GE Healthcare®) to quantify fat mass and lean mass using CoreScan™ software. All dual‐energy x‐ray absorptiometry measurements were carried out by the same trained technician.

### Muscular strength rest (1‐RM) and functionality

Before testing, all patients performed two familiarization sessions separated by at least 72 h. Lower‐ and upper‐limb maximal dynamic strength was assessed in the leg‐press (45°) and bench‐press, respectively (Nakagym, São Paulo, Brazil). Coefficient of variation for 1RM in the leg‐press and bench‐press were 2.8% and 1.1%, respectively.

Functionality was measured by the timed‐stands test and the timed‐up‐and‐go test. The timed‐stands test evaluates the maximum number of stands that an individual can perform from a standard‐height armless‐chair (i.e. 45 cm), whereas the timed‐up‐and‐go test registers the minimal time (in seconds) that each individual requires to rise from a standard chair, walk to a line on the floor 3 m away, turn around, and sit down again. Two attempts were allowed for each test. All tests were conducted by the same investigator to avoid bias. Coefficient of variation for timed‐stands test and timed‐up‐and‐go test were 2.1% and 3.5%, respectively.

### Physical activity monitoring

Physical activity levels and sedentary behaviour were objectively measured using ActiGraph GT3X® accelerometers (ActiGraph, Pensacola, Florida). Patients were instructed to wear the accelerometer for seven consecutive days during waking hours, except when bathing. Physical activity was assessed during non‐training periods [i.e. at PRE, before the commencement of exercise intervention (POST3), and after the completion of the exercise intervention (POST9)]. All patients were required to accumulate at least 10 h of valid activity recordings per day for at least five days. The accelerometer was worn on an elastic belt at the waistline on the right side of the hip. To accurately register when the device was worn and removed, patients kept a daily log. Data were exported from the device in 60 s epochs, using ActiLife 6 software (ActiGraph, Pensacola, Florida). Non‐wear periods were defined as intervals of at least 60 min of zero activity counts, assuming a tolerance of no more than 2 min of counts between 0 and 100. Freedson *et al*.^26^ cut‐points were used to define epochs as follows: sedentary time (<100 counts per minute), light‐intensity physical activity (≥100 to <1952 counts per minute), and moderate‐to‐vigorous physical activity (≥1952 counts per minute). Sedentary behaviour and moderate‐to‐vigorous physical activity data are presented as minute per day.

### Muscle biopsies

All percutaneous muscle biopsy procedures were performed by a trained physician. The patients assumed a comfortable reclining position with both legs outstretched. The *vastus lateralis* biopsy site, (at a point 10–15 cm proximal from the tibial tuberosity and 5 cm lateral from the midline of the femoral course), was prepared with chlorhexidine gluconate solution. Skin and subcutaneous tissue were infiltrated with a 3 mL lidocaine hydrochloride 2% *w*/*v* solution, ensuring that the *fascia lata* was well infiltrated. After ensuring adequate local anaesthesia, an incision was made in the lateral thigh at the biopsy site directly through the overlying skin, subcutaneous fat, and *fascia lata*. A 5 mm modified Bergström needle was then inserted through the fascia, and an assistant immediately applied suction using a 60 mL syringe connected to a canister and attached to the top of the needle. A muscle sample was removed, and the skin was closed with skin closure tape. To minimize risk of infection and bruising, a pressure dressing was immediately applied and maintained for 72 h. All post‐intervention biopsies were performed 72 h following the last exercise session at an adjacent distal incision to the baseline site, approximately 0.5 cm apart. There were no adverse events related to the procedure.

### Immunohistochemistry

Frozen muscles were embedded in optimal cutting temperature compound and were sectioned (7 μm thick) on a cryostat. For fibre immunostaining assay, muscle samples were brought to room temperature and fixed in methanol for 10 min, washed [three 10 min washes with phosphate‐buffered saline (PBS)], then blocked for 60 min in a blocking buffer solution (containing 1% PBS, 5% bovine serum albumin, and 0.3% Triton X‐100). The slides were then incubated with primary antibody overnight at 4°C. The next day, slides were again washed (three 10 min washes with PBS) and incubated in appropriate secondary antibody in the dark for 1 h at room temperature. Nuclei were labelled using fluoroshield mounting medium with DAPI (Ab104139, Abcam), prior to cover slipping slides. Images were captured with an Olympus BX51 Fluorescence microscope with a magnification of ×20. For Type I and Type II fibre cross‐sectional area (fCSA), myonuclei and satellite cell content (SC), a minimum of 120 fibres per subject at each time point was analysed.^27^ The quantification of muscle fibre capillaries [capillary contacts (CC, the number of capillaries around a fibre), capillary‐to‐fibre ratio on an individual fibre basis (C/Fi), and capillary‐to‐fibre perimeter exchange index (CFPE, an estimate of the capillary‐to‐fibre surface area)] were performed on 50 muscle fibres/subject/time point, as previously described.^28^ All immunohistochemical images were analysed by a single researcher in a blinded manner. Coefficient of variation between two blinded measurements were 3.7%, 3.5%, 3.7%, 3.3%, 3.3%, 4.4%, 2.6%, and 3.4% for Types I and II muscle fCSA, Types I and II muscle fibre myonuclei content, Types I and II muscle fibre myonuclear domain, CC, C/Fi, CFPE, and SC, respectively.

### RNA‐sequencing and bioinformatics analysis

For RNA sequencing (RNA‐seq), muscle RNA was isolated (six patients per group) using the RNeasy Fibrous Tissue Mini Kit (Qiagen®). Total RNA concentration was assessed spectrophotometrically at 260 nm (GE Healthcare®) and RNA integrity number (RIN ≥ 7) was checked using a Bioanalyzer 2100 (Agilent®). RNA library construction and sequencing were performed in the Genomics Core at Pennington Biomedical Research Center. Sample concentration was normalized and cDNA pools were created for each sample, and subsequently tagged with a barcoded oligo adapter to allow for sample specific resolution using the Quant‐seq® 3′ mRNA‐Seq Library Prep Kit (Lexogen®). Sequencing was carried out using an Illumina Seq 2500 platform (Illumina®) with 75 bp single end reads. Reads were aligned to the human‐reference genome (GRCh38.77). Annotation and preparation of the sequencing data for downstream analysis was performed by custom *R* scripts^29^ with annotations downloaded from BioMart (R.V3.4.3, Rstudio V1.1.423 and biomaRt V2.24.1). Quant‐Seq raw data are available in the NCBI GEO database (accession GSE 137631).

Raw count data were normalized via TMM normalization in edgeR^30^ and adjusted for mean–variance effects via the voom function in limma.^31^ Differential gene expression analysis was performed via the limma empirical Bayes analysis pipeline by adjusting the model design matrix using paired or unpaired analysis, depending upon the specific contrasts. Genes with a false discovery rate (FDR) ≤ 0.05 were considered as differentially expressed. Pathway enrichment analysis was carried out via the gene set enrichment analysis (GSEA) pre‐ranked method by querying a ‘custom’ database consisting of gene‐sets from Wikipathways (www.wikipathways.org) plus user‐defined gene sets corresponding to specific biological functions. Pathway enrichments with an FDR ≤ 10% and the absolute normalized enrichment score (NES) ≥ 1.5 were considered significant.

### Quantitative real‐time PCR

Total muscle RNA was lysed in 1 mL of TRIzol reagent (Invitrogen®) and RNA was isolated using standard techniques. The Superscript Platinum One‐Step kit (Invitrogen®), with incorporated Maxima SYBR Green/ROX qPCR Master Mix (Thermo Fisher Scientific®) on a Step One™ Real‐Time PCR System (Applied Biosystems®) was used to perform the qRT‐PCR experiments. All primers were purchased from Invitrogen®. The fold changes from PRE were calculated using the 2^*−ΔΔCt*^ method,^32^ and all mRNA levels were normalized to beta‐2‐microglobulin (*β2M*).

### Western blotting

Protein extraction followed by quantification was performed via Western blotting as described previously.^33^ In brief, cell lysates (30–40 μg) were resolved by sodium dodecyl polyacrylamide gel electrophoresis, electrophoresis to PVDF membrane, and incubated overnight with appropriate primary antibodies followed by incubation in appropriate HRP tagged secondary antibody. Signals were revealed by enhanced chemiluminescence ECL reagent (SuperSignal™ West Femto Substrate, Thermo Fisher Scientific). Gel‐to‐gel variation and equal protein loading were controlled using a standardized sample on each gel. Results were corrected to Ponceau red staining (0.5%, w:v) of the membrane for all immunoblot assays.^34^ Blots were quantified using the Image‐J software®. Details of all reagents and resources are provided in supporting information, *Table* S1.

### Sample size calculation and statistical analysis

Sample size was determined with the aid of a G‐Power software (version 3.1.2—*Universitat Kiel*, Germany) package. The analysis was conducted by inputting α error (0.05), power (1 − β error = 0.90), and effect size (Hedges' g = 0.20), considering previous data indicating moderate between‐group effect size (RYGB vs. RYGB + ET) for fat‐free mass in individuals undergoing bariatric surgery.^18^ Calculations were based on an ANOVA with repeated measures (within‐between interactions) and the total sample size was determined to be 56 patients. To account for midtrial withdrawals, the sample was increased by ~40% (i.e. 80 patients).

Data are presented as mean ± standard deviation (SD), estimated mean difference between groups (EMD) at POST9 (only in the presence of group x time interaction), 95% confidence interval (95%CI), unless otherwise indicated. Baseline characteristics were compared between the two groups using either Student's *t* test or Fisher exact test. The data were analysed using an intention‐to‐treat (ITT) approach to preserve the integrity of randomization. Dependent variables were analysed by a mixed model analysis for repeated measures, using group (RYGB and RYGB + ET group) and time (PRE, POST3, and POST9) as fixed factors, and subjects as a random factor using SAS software version 9.1. *Post‐hoc* tests with Tukey's adjustment were performed for multiple comparisons. Additionally, in order to verify efficacy of the exercise training in body composition parameters, *independent t tests* were performed to test possible between‐group differences in absolute changes (∆) between POST9 to PRE. To compare experimental groups (RYGB vs. RYGB + ET) at POST9 with reference values from healthy women lean control group, values of muscle strength, functionality, capillarization, and SC content were transformed into standardized scores (i.e. *Z*‐score) having mean ± SD values of the CTRL as reference for each assessment. In this analysis it is assumed that the closer the *Z*‐scores are to zero the more comparable they are to the healthy women in the lean control group. In addition, in order to statistically compare the *Z*‐score of each dependent variable of the non‐exercise and exercise group with women in the healthy lean control group, a one‐way ANOVA was performed, and in case of *F*‐value significant, *post‐hoc* tests with *Dunnett*'s adjustment were performed for multiple comparisons. Significance level was set at *P ≤* 0.05. To facilitate the reader, main effects of time are described as effects of surgery (POST3‐PRE), whereas group × time interactions at POST9 are described as effects of exercise, unless otherwise stated.

## Results

### Flow of participants

The flow of participants through the study is depicted in *Figure* S1. Three‐hundred and thirty‐eight patients were screened for participation. Eighty patients met inclusion criteria and were randomly (computer‐generated randomization code) assigned (1:1) to either RYGB (*n* = 40) or RYGB + ET (*n* = 40). Thirteen patients dropped out of the RYGB group (did not perform surgery: *n* = 9; personal reasons: *n* = 4) and 12 dropped out from the RYGB + ET group before the intervention (did not perform surgery: *n* = 7; personal reasons: *n* = 5). Patients from the RYGB + ET group attended 83 ± 11% of the exercise training sessions. Both groups were comparable regarding baseline characteristics (*Table* 1).

**Table 1:** Baseline characteristics of the participants

Outcomes	RYGB (*n* = 40)	RYGB+ET (*n* = 40)	*P* value	Healthy lean control group (*n* = 20)
Age, years	42 ± 8	38 ± 7	0.0719	38 ± 6
Body composition				
Body mass, kg	124.6 ± 21.4	127.0 ± 22.0	0.6159	63.1 ± 5.5
BMI, kg/m^2^	47.4 ± 7.6	48.9 ± 6.5	0.3533	21.8 ± 1.4
Fat mass, kg	62.9 ± 14.9	65.5 ± 14.2	0.4314	15.6 ± 3.0
Fat‐free mass, kg	56.6 ± 6.9	56.2 ± 7.2	0.8504	41.4 ± 6.5
Lower‐limb fat‐free mass, kg	20.9 ± 3.8	21.5 ± 3.5	0.5237	14.0 ± 1.6
Upper‐limb fat‐free mass, kg	6.4 ± 0.8	6.1 ± 1.1	0.2396	4.3 ± 0.9
Medication				
β‐blockers, *n* (%)	5 (12.5)	4 (10.0)	>0.9999	‐
Metformin, *n* (%)	5 (12.5)	6 (15.0)	>0.9999	‐
Insulin, *n* (%)	5 (12.5)	2 (5.0)	0.4315	‐
ACE inibitor, *n* (%)	5 (12.5)	7 (17.5)	0.7555	‐
Calcium channel blocker, *n* (%)	1 (2.5)	2 (5.0)	>0.9999	‐
Angiotensin II receptor antagonism, *n* (%)	9 (22.5)	8 (20.0)	>0.9999	‐
Diuretics, *n* (%)	10 (25.0)	11 (27.5)	>0.9999	‐
Muscle function				
1‐RM leg‐press, kg	190 ± 60	201 ± 56	0.4337	177 ± 37
1‐RM leg‐press, kg/body mass	1.5 ± 0.49	1.6 ± 0.5	0.6283	2.9 ± 0.5
1‐RM bench‐press, kg	36 ± 8	38 ± 8	0.4198	33 ± 12
1‐RM bench‐press, kg/body mass	0.3 ± 0.1	0.3 ± 0.1	0.7625	0.5 ± 0.2
Timed‐up‐and‐go, s	7.6 ± 1.1	7.4 ± 1.0	0.6339	4.7 ± 0.4
Timed‐stands, reps	13 ± 2	14 ± 2	0.0820	22 ± 2
Physical activity level				
Sedentary behaviour, min/day	538 ± 110	588 ± 162	0.3006	587 ± 82
Sedentary behaviour, %/day	65 ± 10	67 ± 10	0.5560	67 ± 5
MVPA, min/day	26 ± 13	37 ± 28	0.1745	57 ± 22
MVPA, %/day	3 ± 2	4 ± 3	0.2170	6 ± 2
Cardiorespiratory fitness				
VAT, mL/kg/min	8.6 ± 1.72	9.6 ± 2.4	0.2020	16.5 ± 5.2
RCP, mL/kg/min	13.8 ± 2.21	14.0 ± 2.4	0.6839	27.5 ± 6.6
VO_2 peak_, mL/kg/min	15.5 ± 2.7	15.8 ± 2.3	0.5453	33.2 ± 6.8

Data are expressed as mean ± SD or number of patients (%). 1‐RM: maximal dynamic strength; ACE, angiotensin‐converting enzyme; MVPA, moderate‐to‐vigorous physical activity; RCP, respiratory compensation point; RYGB + ET: Roux‐en‐Y gastric bypass plus exercise training group; RYGB: Roux‐en‐Y gastric bypass plus non‐exercise group; VAT, ventilatory anaerobic threshold; VO_2 peak_, peak oxygen uptake.

### Changes in body composition

**Figure 1:**
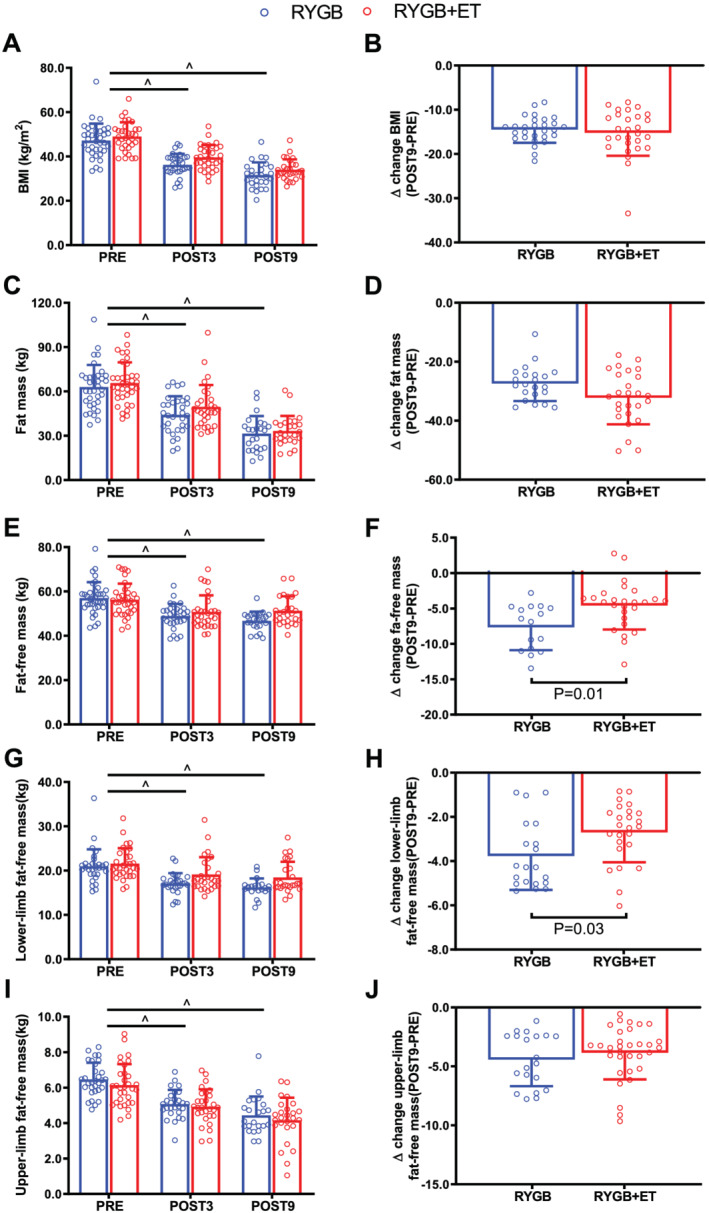
Changes in body composition. RYGB + ET (*n* = 28): Roux‐en‐Y gastric bypass plus exercise training group; RYGB (*n* = 27): Roux‐en‐Y gastric bypass plus non‐exercise. Body mass index (BMI), fat mass, fat‐free mass, and lower‐ and upper‐limb fat‐free mass in the experimental groups (Panels *A, C, E, G*, and *I*, respectively). Absolute changes (∆) from POST9 to PRE for BMI, fat mass, fat‐free mass, lower‐limb fat‐free mass (Panels *B, D, F, H*, and *J*, respectively). Data are expressed as mean ± SD. PRE, before surgery (baseline); POST3, 3 months following surgery; POST9, 9 months following surgery. ^ indicates *P* < 0.05 for main effect of time.

The BMI, fat mass, total fat‐free mass, and lower‐limb and upper‐limb fat‐free mass were reduced at 3 and 9 months after surgery (main effect of time: all *P* < 0.0001) (*Figure* 1, Panels A, C, E, and G). Additionally, delta analysis (POST9‐PRE) revealed that reductions in total (RYGB: ∆: −7.9 kg; RYGB + ET: ∆: −4.9 kg; between group comparison: ∆: −3.0 kg; 95% CI: −4.54 to −1.60; *P* = 0.0042) and lower‐limb fat‐free mass (RYGB: ∆: −3.8 kg; RYGB + ET: ∆: 2.7 kg; between group comparison: ∆: −1.1 kg; 95%CI: −0.47 to −1.77; *P* = 0.0268) were greater in RYGB in comparison to RYGB + ET (*Figure* 1, Panels B, D, F, and H, respectively), indicating that exercise training preserved total and lower‐limb fat‐free mass in the exercised group. In contrast, delta analysis (POST9‐PRE) did not detect between‐group difference for upper‐limb fat‐free mass (RYGB: ∆: −1.7 kg; RYGB + ET: ∆: −1.7 kg; between group comparison: ∆: 0.0 kg; 95% CI: −0.54 to 0.54; *P* = 0.9556).

### Muscular strength, functionality, and physical activity level

**Figure 2:**
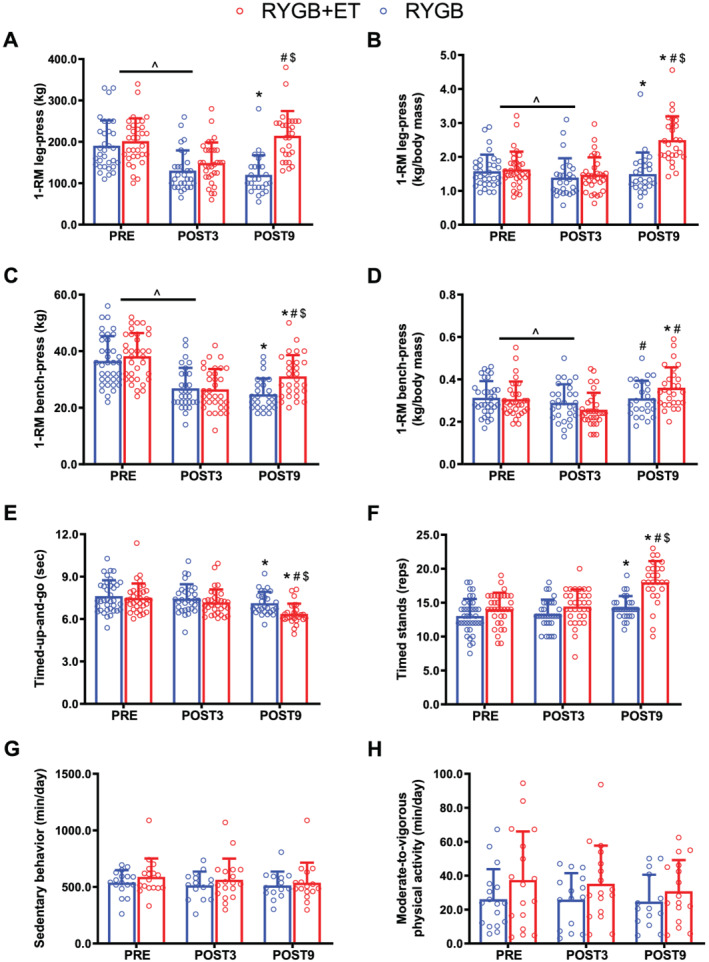
Muscular strength, functionality, and physical activity level. RYGB + ET (*n* = 28): Roux‐en‐Y gastric bypass plus exercise training group; RYGB (*n* = 27): Roux‐en‐Y gastric bypass plus non‐exercise. Absolute and relative lower‐limb (Panels *A* and *B*, respectively) and upper‐limb (Panels *C* and *D*, respectively) strength performance in the 1‐RM test. Functionality in the timed‐up‐and‐go test and timed‐stand test (Panels *E* and *F*, respectively). Time spent in sedentary behaviour and moderate‐to‐vigorous physical activity level (Panels *G* and *H*, respectively). Data are expressed as mean ± SD. PRE: before surgery (baseline); POST3: 3 months following surgery; POST9: 9 months following surgery; ^ indicates *P* < 0.05 for main effect of time. * indicates *P* < 0.05 in comparison with PRE; ^#^ indicates *P* < 0.05 in comparison with POST3; ^$^ indicates *P* < 0.05 for between‐group comparison with POST9.

There were significant decreases in absolute and relative lower‐ and upper‐limb muscle strength following surgery (main effect of time: both *P* < 0.0001) (*Figure* 2, Panels A–D). Importantly, the RYGB+ET group showed absolute and relative lower‐limb muscle strength (EMD: 83.2 kg; 95% CI: 43.2 to 122.8; *P* < 0.0001; EMD: 0.9 kg/body mass, 95% CI: 0.4 to 1.3; *P* < 0.0001, respectively) and absolute upper‐limb muscle strength (EMD: 5.4 kg; 95% CI: 0.03 to 10.9, *P* = 0.0529) than the RYGB group at POST9 (*Figure* 2, Panels A–D). Functional tests, including the timed‐up‐and‐go and timed‐stands scores improved 9 months following surgery in both groups (RYGB + ET: both *P* < 0.0001; RYGB: *P* = 0.0008 and *P* = 0.012, respectively); however, the RYGB + ET group performed better than the RYGB group in timed‐up‐and‐go (EMD: 0.2 s, 95% CI: −0.1 to 1.4, *P* = 0.0566) and timed‐stands tests (EMD: 3.5 repetitions, 95% CI: 1.7 to 5.3, *P* < 0.0001, respectively) (*Figure* 2, Panels E and F). Physical activity levels were unchanged in both groups (all *P* > 0.05) (*Figure* 2, Panels G and H).

### Muscle fibre cross‐sectional area, myonuclei content, myonuclear domain, muscle fibre capillarization, and satellite cell content

**Figure 3:**
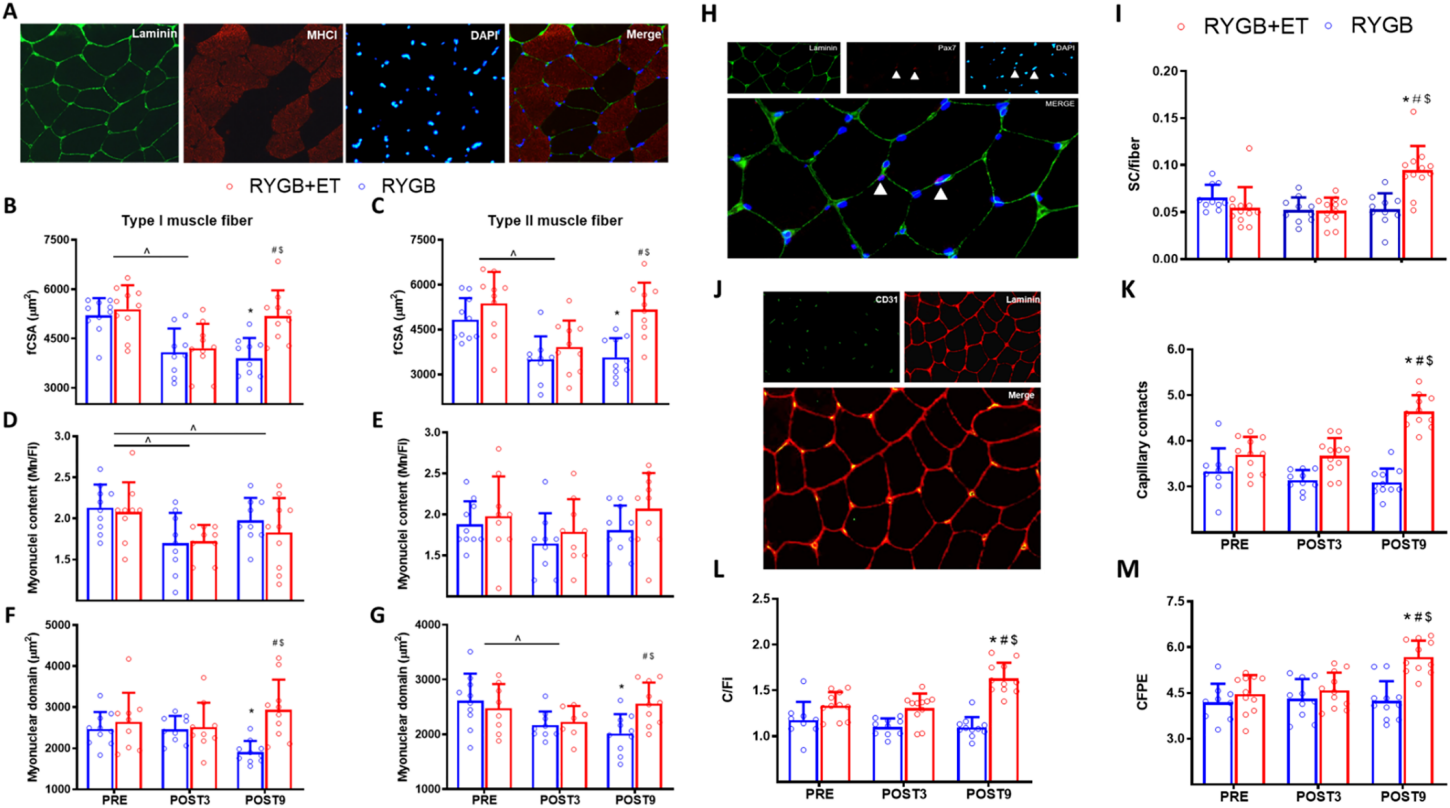
Muscle fibre cross‐sectional area, myonuclei content, myonuclear domain, muscle fibre capillarization, and satellite cell content. RYGB + ET (*n* = 11): Roux‐en‐Y gastric bypass plus exercise training group; RYGB (*n* = 11): Roux‐en‐Y gastric bypass plus non‐exercise. A representative image of the immunofluorescence staining for analysis of Types I and II muscle fibre cross‐sectional area (fCSA), myonuclei content, myonuclear domain (Panel *A*), satellite cells content (Panel *H*), and capillarization (Panel *J*). Types I and II muscle fibre cross‐sectional area (fCSA) (Panels *B* and *C*, respectively), myonuclei content (Panels *D* and *E*, respectively), myonuclear domain (Panels *F* and *G*, respectively), satellite cells content (Panel *I*), capillary contacts (CC) (Panel *K*), capillary‐to‐fibre ratio on an individual fibre basis (C/Fi) (Panel *L*), and capillary‐to‐fibre perimeter exchange index (CFPE) (Panel *M*). Data are expressed as mean ± SD. PRE: before surgery (baseline); POST3: 3 months following surgery; POST9: 9 months following surgery. ^ indicates *P* < 0.05 for main effect of time; * indicates *P* < 0.05 in comparison with PRE; ^#^ indicates *P* < 0.05 in comparison with POST3; ^$^ indicates *P* < 0.05 for between‐group comparison with POST9.

To further examine the effect of exercise on muscle remodelling, we performed immunostaining assays from *vastus lateralis* samples to assess adaptations at the muscle fibre level, specifically Type I and Type II muscle fCSA, satellite cell content, and capillarization (*Figure* 3, Panels A, H, and L, respectively). Type I and Type II fCSA decreased 3 months following surgery in both groups (main effect of time: both *P* < 0.0001). The RYGB + ET group showed greater Type I fCSA (EMD: 1289.2 μm^2^, 95% CI: 355.7 to 2222.6, *P* = 0.0025) and Type II fCSA (EMD: 1600.1 μm^2^, 95% CI: 468.4 to 2731.8, *P* = 0.0019) than the RYGB group (*Figure* 3, Panels B and C) at POST9. Of relevance, Types I and II fCSA in the RYGB + ET reached pre‐surgery levels (*P* > 0.05).

The satellite cell pool was increased after the intervention in the RYGB + ET group, whereas no changes were noted in the RYGB group across time (both *P* > 0.05). Of relevance, the RYGB + ET group had a larger satellite cell pool than the RYGB group after the intervention (EMD: 0.04 satellite cell/fibre; 95% CI: 0.01 to 0.06; *P* < 0.0001) (*Figure* 3, Panel I). Both groups demonstrated reduced Type I muscle fibre myonuclei content at POST3 and POST9 vs. PRE (*P* < 0.0001 and *P* = 0.0308, respectively).

The RYGB + ET group increased the expression of the angiogenesis markers *ANGP1*, *ANGP2*, *TEK*, and *VEGF* (EMD: 2.2 fold change; 95% CI: 0.4 to 4.1; *P* < 0.0071; EMD: 4.0 fold change; 95% CI: 2.8 to 5.3; *P* < 0.0001; EMD: 1.6 fold change; 95% CI: 0.7 to 2.6; *P* < 0.0001; EMD: −241.5 fold change; 95% CI: −438.9 to −44.2; *P* < 0.0083, respectively) (*Figure* S2, Panels B, C, E, and G, respectively). Accordingly, the RYGB + ET group showed greater muscle fibre capillarization (assessed by CC, C/Fi, and CFPE) than the RYGB group at POST9 (EMD: 1.5 capillary contacts; 95% CI: 1.0 to 2.0; *P* < 0.0001; EMD: 0.5 C/Fi; 95% CI: 0.3 to 0.7; *P* < 0.0001; EMD: 1.4 CFPE; 95% CI: 0.6 to 2.2; *P* < 0.0001) (*Figure* 3, Panels K, L, and M, respectively).

### Pathway enrichment analysis related to muscle plasticity, gene, and protein expression of muscle remodelling pathways

**Figure 4:**
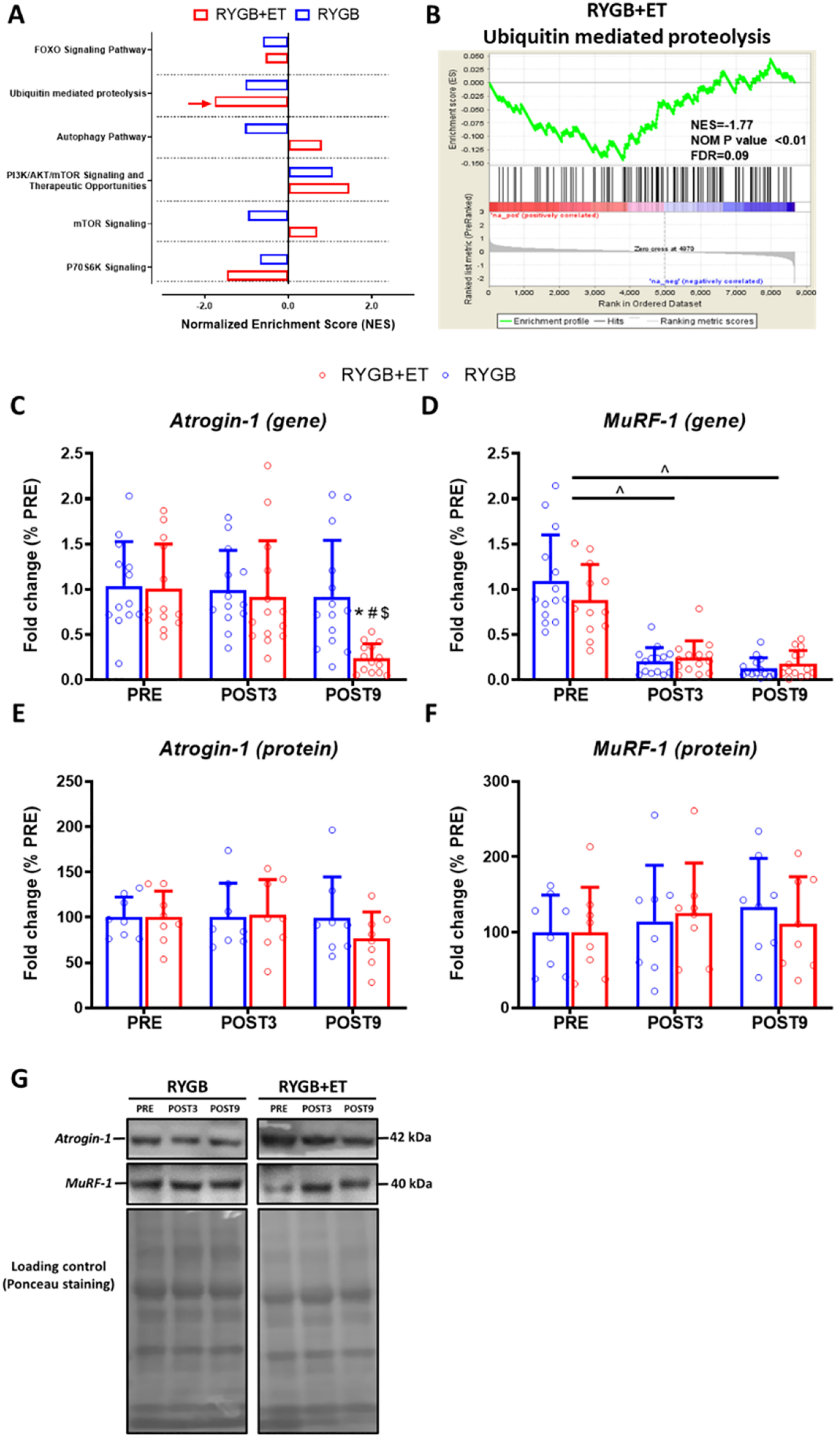
Pathway enrichment analysis related to muscle plasticity, gene and protein expression of atrogin‐1 and MuRF‐1. RYGB+ET [*n* = 14(8 for western blot)]: Roux‐en‐Y gastric bypass plus exercise training group; RYGB [*n* = 14 (8 for western blot)]: Roux‐en‐Y gastric bypass plus non‐exercise. Gene set enrichment analysis (GSEA) related to muscle protein synthesis and breakdown (Panel *A*) and the GSEA‐plot of the ubiquitin mediated proteolysis pathway (Panel *B*). Gene and protein expression for *Atrogin‐1* (Panels *C* and *E*, respectively) and *MuRF‐1* (Panels *D* and *F*, respectively). Representative image of the Western blot bands for *Atrogin‐1* and *MuRF‐1* (Panel *H*). Data are expressed as mean ± SD. Red arrow indicates an enrichment with a false discovery rate (FDR) ≤ 10% and the absolute log2 fold‐change >1.5; PRE: before surgery (baseline); POST3: 3 months following surgery; POST9: 9 months following surgery. ^ indicates *P* < 0.05 for main effect of time; * indicates *P* < 0.05 in comparison to PRE; ^#^ indicates *P* < 0.05 in comparison to POST3; ^$^ indicates *P* < 0.05 for between‐group comparison at POST9.

Gene set enrichment analysis revealed that the *ubiquitin‐mediated proteolysis* pathway was significantly suppressed in the RYGB + ET group at POST9 vs. PRE (NES: −1.7; *P* < 0.01; FDR: 0.09), whereas no changes were observed in the RYGB group (*Figure* 4, Panels A and B, respectively). Supporting the RNA‐seq data, *Atrogin‐1* gene expression was lower in the RYGB + ET vs. RYGB group (EMD: −1.9‐fold change; 95% CI: −3.0 to −0.8; *P* < 0.0001) (*Figure* 4, Panel C).

Our data indicated a decreased expression of the autophagy system‐related genes *BNIP3* and *CTSL1* at 3 and 9 months after the surgery, independent of exercise (both *P* < 0.0001) (*Figure* S3, Panels A and C, respectively).

*mTOR* pathway‐related proteins, *p‐4E‐BP1/4E‐BP1* was reduced at POST9 and POST3 vs. PRE, independent of exercise (main effect of time: *P* = 0.0125 and *P* = 0.0412, respectively) (*Figure* S4, Panel I). However, no significant changes were observed in *mTOR* and *P70S6K* because of surgery and exercise (*Figure* S4, Panels A–H).

### Muscle genotype and phenotype of post‐bariatric patients in comparison with healthy lean controls

**Figure 5:**
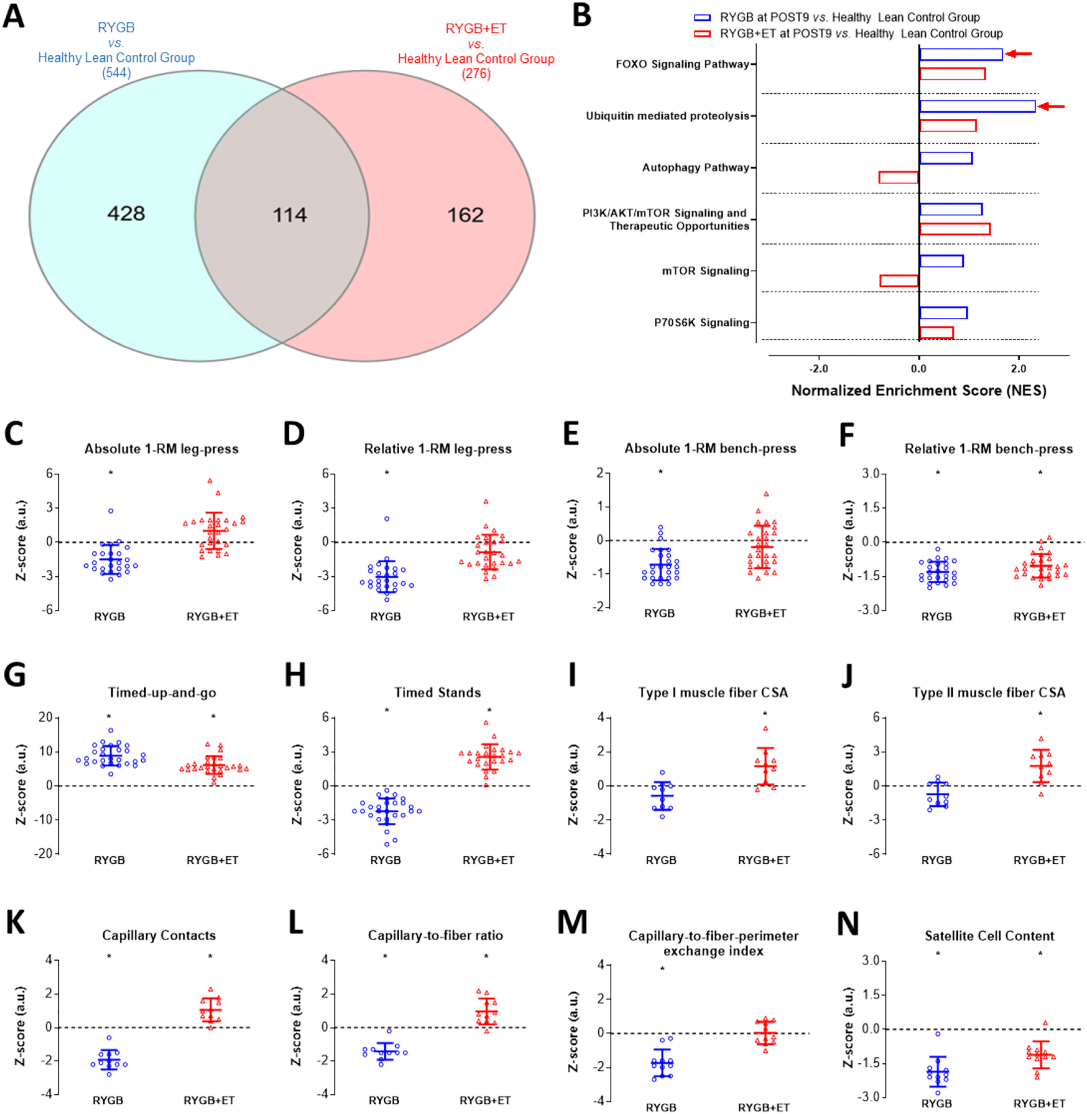
Normalized skeletal muscle cell signalling pathways and physiological outcomes. RYGB + ET: Roux‐en‐Y gastric bypass plus exercise training group; RYGB: Roux‐en‐Y gastric bypass plus non‐exercise. Venn diagram of the total number of differentially expressed genes (FDR ≤ 0.05), and gene set enrichment analysis (GSEA) related to muscle protein synthesis and breakdown in the RYGB + ET and RYGB groups at POST9 in comparison with healthy lean control group (Panels *A* and *B*, respectively). Comparison of the RYGB + ET and RYGB groups at POST9 with reference values from healthy lean control group: *Z*‐score for absolute and relative lower‐limb (Panels *C* and *D*, respectively) and upper‐limb (Panels *E* and *F*, respectively) strength performance in the 1‐RM test, functionality in the timed‐up‐and‐go test and timed‐stand test (Panels *G* and *H*, respectively), Types I and II muscle fibre cross‐sectional area (CSA) (Panels *I* and *J*, respectively), muscle fibre capillarization parameters (Panels *K*, *L*, and *M*, respectively) and satellite cell content (Panel *N*). Data are expressed as mean ± SD. Red arrow indicates an enrichment with a false discovery rate (FDR) ≤ 10% and the absolute log2 fold‐change >1.5; PRE: before surgery (baseline); POST3: 3 months following surgery; POST9: 9 months following surgery. * indicates *P* < 0.05 in comparison with healthy lean control group.

To investigate the extent to which exercise may rescue muscle genotype and phenotype in patients who underwent bariatric surgery, we compared the RNA‐seq data from RYGB + ET and RYGB groups (at POST9) vs. healthy lean control group. In comparison with controls, the RYGB + ET group showed a much lower number of differentially expressed genes (276 genes) than the RYGB group (544 genes), with genes at FDR ≤ 0.05 being defined as differentially expressed. (*Figure* 5, Panel A). Interestingly, GSEA did not show any significant difference between the RYGB + ET group and controls (all *P* > 0.05), whereas pathways related to the ubiquitin‐proteasome system (i.e. *FOXO signalling pathway and ubiquitin‐mediated proteolysis*) were up‐regulated in the RYGB group vs. controls (*FOXO signalling pathway*: NES: 1.68, FDR: 0.2117, *P* = 0.0316; *ubiquitin proteolysis pathway*: NES: 2.34, FDR: 0.0545, *P* = 0.0020) (*Figure* 5, Panel B).

To further examine whether skeletal muscle phenotype also follows the same pattern, muscle strength, functionality, fCSA, capillarization, and SC, data were transformed into standardized scores (i.e. *Z*‐score), and then the RYGB + ET and RYGB groups were compared with controls. The RYGB + ET group and controls showed similar values in absolute and relative lower‐limb muscle strength and absolute upper‐limb muscle strength (*P* = 0.0531, *P* = 0.0620, *P* = 0.9999, respectively). In contrast, the RYGB group exhibited reduced values of absolute and relative lower‐limb muscle strength and absolute upper‐limb muscle strength vs. controls (*P* = 0.0002, *P* < 0.0001, *P* = 0.0171, respectively) (*Figure* 5, Panels C–F). The RYGB + ET group demonstrated even faster scores in the timed‐stand test than controls (*P* < 0.0025), while the RYGB group showed slower scores than controls (*P* < 0.0001). Both groups exhibited lower performance in the relative upper‐limb muscle strength test and timed‐up‐and‐go test as compared with controls (*P* < 0.05 for all) (*Figure* 5, Panels F and G, respectively).

The RYGB + ET group also showed higher Types I and II fCSA, CC, and C/Fi, and comparable values of CFPE vs. controls (*P* = 0.0229 and *P* = 0.0038, *P* = 0.0080, *P* = 0.0143, and *P* = 0.9871, respectively). Conversely, the RYGB group had comparable type I and II fCSA, and lower values of CC, C/Fi, and CFPE *vs*. controls (*P* = 0.3341, *P* = 0.2975, *P* < 0.0001, *P* = 0.0004, and *P* < 0.0001, respectively) (*Figure* 5, Panels I–M). Both groups showed lower SC content than controls (*P* = 0.0035 and *P* < 0.0001, respectively) (*Figure* 5, Panel N).

## Discussion

This trial demonstrated that a 6 month, three‐times‐a‐week, supervised exercise training programme attenuated the loss of fat‐free mass and reversed the loss of muscle strength among women who had bariatric surgery. The exercise training programme completely reversed surgery‐induced muscle fibre atrophy, and this was accompanied by increases in capillarization and satellite cell content. Interestingly, RNA‐seq and RT‐PCR analyses support the hypothesis that exercise suppressed the ubiquitin‐proteasome system, which may partially explain the beneficial effect of exercise on muscle remodelling. Of relevance, genotype and phenotype data revealed that skeletal muscle of patients who exercised resembled that of healthy lean individuals.

Bariatric surgery is the treatment of choice for severe obesity, but this approach is not free of adverse effects. In consonance with previous studies,^3^, ^4^ we observed a drastic decrease in the absolute and relative lower‐limb and upper‐limb muscle strength 3 months following surgery, whereas no significant change was observed in functionality and relative strength. In contrast, the addition of a post‐surgery exercise training programme demonstrated to be an effective strategy to reverse the loss of absolute and relative lower‐limb muscle strength and relative upper‐limb muscle strength following surgery. It is noteworthy that exercised patients also increased absolute upper‐limb muscle strength following intervention, but it did not return to baseline values. Moreover, the combination of surgery plus exercise training provided improvements in functionality to a greater extent than that seen with surgery/standard of care 9 months following intervention. These data corroborate the hypothesis that exercise can offset the decline in muscle strength and function that is associated with bariatric surgery.^16^, ^17^ Conversely, physical activity levels were unchanged in both groups throughout the study, supporting the idea that neither bariatric surgery nor structured exercise training are effective to promote profound increases in unstructured physical activity levels in this population.^35^

Muscle atrophy and dysfunction following bariatric surgery is partially attributed to a substantial decrease in fat‐free mass.^16^, ^17^, ^18^ In agreement with previous studies, BMI, fat mass, total fat‐free mass, and lower‐limb and upper‐limb fat‐free mass were reduced at 3 and 9 months after surgery. While lifestyle interventions (e.g. exercise training and nutrition) are recommended to attenuate the loss of fat‐free mass among patients undergoing bariatric surgery,^36^, ^37^ efficacy is still debated.^16^, ^17^, ^18^ In this study, the losses in total and lower‐limb fat‐free mass were attenuated in the RYGB + ET in comparison with the RYGB group, supporting previous findings.^16^, ^17^ Of note, the benefit of exercise on total and lower‐limb fat‐free mass was independent of the relatively low protein consumption reported by the patients (i.e. <1 g·kg^−1^·day^−1^) (*Table* S2), suggesting that exercise *per se* is an important anabolic stimulus for this population, although the between‐group differences for total and lower‐limb fat‐free mass were relatively small. Contrary to total and lower‐limb fat‐free mass data, exercise training was not able to mitigate upper‐limb fat‐free mass loss, despite the observed increase in upper‐limb muscle strength. A possible explanation may be related to the effects of aerobic exercise on muscle mass in untrained individuals.^38^ In this context, it is possible to speculate that the aerobic exercise (treadmill) may have provided an additional anabolic stimulus to the lower‐limb but not to the upper‐limb muscles, especially considering that patients still presented with high body mass values, even after surgery (BMI ~ 36 kg/m^2^). Gains in upper‐limb muscle strength may be linked to neural adaptations such as increase in the firing rate, inter‐ and intra‐ muscular coordination, motor unit synchronic improvement.^39^

To further examine the effect of exercise on muscle remodelling at the muscle fibre level, we performed immunostaining assays from *vastus lateralis* samples. Our findings demonstrate a decrease in Type I and Type II fCSA 3 months following surgery in both groups. In contrast, the addition of a post‐surgery exercise training routine increased Type I and Type II fCSA when compared with the non‐exercised group. Furthermore, exercise was able to fully reverse the muscle fibre atrophy induced by surgery, as Types I and II fCSA were increased back to pre‐surgery levels in RYGB + ET group.

Muscle fibre remodelling depends on the pool of satellite cells, which are localized between the basal lamina and sarcolemma. When activated, satellite cells proliferate and differentiate to supply additional myonuclei to the fibre or replenish the resident pool of satellite cells.^40^ We observed that exercise training increased the pool of satellite cells after the intervention, but muscle fibre myonuclei content remained unchanging, challenging the myonuclear domain hypothesis, according to which, during the hypertrophic process, myonuclei accretion is required to maintain a given surrounding territory of cytoplasm under the control of a given number of myonuclei. In another perspective, satellite cells are also thought to regulate fibrogenic cell collagen expression and, hence, muscle fibre extracellular matrix remodelling, another key factor related to muscle fibre remodelling.^41^ In this regard, we recently demonstrated that bariatric surgery decreased muscle extracellular matrix deposition, an effect that was potentiated by exercise.^25^ Taken together, these findings suggest that satellite cells may have been predominantly driven towards skeletal muscle extracellular matrix remodelling, rather than myonuclear accretion.

The delivery of molecular signals [e.g. insulin‐like growth factor‐1 (IGF‐1), hepatocyte growth factor (HGF), interleukin‐6 (IL‐6) and myostatin] to skeletal muscle cells is highly dependent on muscle fibre capillarization, a key aspect in muscle remodelling.^42^ The formation of new capillaries in myofibres depends on a complex process termed angiogenesis, which is regulated by anti‐angiogenic and pro‐angiogenic factors. Our data indicate that exercise increased the expression of different angiogenesis markers (i.e. *ANGP1*, *ANGP2*, *TEK*, and *VEGF*), which was accompanied by increased muscle fibre capillarization. Collectively, these findings suggest that exercise increased muscle fibre perfusion via increased capillarization (mediated by alteration in the expression of numerous angiogenesis markers), possibly resulting in a more efficient delivery of oxygen, nutrients, and growth factors necessary for muscle fibre growth.

To gather mechanistic insights on the effects of surgery combined with exercise on muscle remodelling‐related pathways, we performed RNA‐seq and RT‐PCR analysis of muscle samples that revealed that the *ubiquitin‐mediated proteolysis* pathway and *Atrogin‐1* gene expression was suppressed in the RYGB + ET group at POST9 vs. PRE, but not in the RYGB group. This suggests that exercise mitigated muscle protein breakdown mediated by the ubiquitin‐proteasome system and may represent an important part of the effect of exercise on muscle size.

The autophagic system is responsible for removing and eliminating unfolded and toxic proteins as well as abnormal and dysfunctional organelles, through the formation of a double membrane vesicle that engulfs portion of the cytoplasm, organelles, glycogen, and protein aggregates, such as those that accumulate within the muscle cells of individuals with obesity.^43^ Of interest, we verified a decrease in the expression of autophagy system‐related genes (i.e. *BNIP3* and *CTSL1*) following surgery, which was not affected by exercise. These findings suggest that bariatric surgery induces a lower autophagic flux, possibly because of the substantial reduction in adiposity, which, in turn, may reduce the number of toxic proteins and dysfunctional organelles. This may represent a new mechanism associated with the beneficial effect of bariatric surgery on decreased systemic inflammation and endoplasmic reticulum stress.

*mTOR* pathway is recognized as a key regulator of muscle anabolism. Previous studies have shown that calorie restriction suppresses the expression of *mTOR* pathway‐related proteins,^44^ while mechanical stimuli are known to activate them.^45^ We were unable to identify significant changes in *mTOR* pathway‐related proteins (i.e. *mTOR* and *P70S6K*) as a result of surgery and exercise, with the exception of *p‐4E‐BP1/4E‐BP1 that* was reduced at POST9 and POST3 in comparison with baseline values. It is possible that the absence of change in these proteins was influenced by the timing of the muscle biopsy, because activation of these proteins appears to be dependent on an acute anabolic stimulus (e.g. protein ingestion or resistance exercise).^44^, ^46^ Therefore, caution should be exercised in concluding that exercise and surgery did not influence the *mTOR* pathway in this study.

Muscle mass is regulated by the coordinated balance between the rates of muscle protein synthesis and muscle protein breakdown.^47^ Our findings suggest a complex network of mechanisms related to muscle fibre growth in patients with obesity undergoing bariatric surgery. Despite cellular adaptations occurring in the absence of chronic adaptations in proteins related to mTOR signalling pathway and in myonuclei content, exercise was shown to mitigate muscle protein breakdown, an observation that is supported by reduced gene expression in ubiquitin‐proteasome system related proteins. The increased muscle fibre capillarization and satellite cell content may also help explain our results, as these adaptations may have positively affected the spatial proximity between satellite cell and capillaries, facilitating the delivery of growth factors, thereby improving the myogenic response to exercise.^42^ Although it is reported that exercise increases myonuclei content during hypertrophy‐oriented conditions,^48^ our data suggest that the existing myonuclei in the exercised patients are capable of sustaining new transcriptional requirements of the muscle fibre in order to promote protein accretion under conditions that are unfavourable to hypertrophy (e.g. negative energy balance). It is difficult to put these novel data into perspective with the literature, as these are the first data to have assessed changes in fat‐free mass and histological and molecular parameters related to muscle fibre adaptations in obese patients undergoing bariatric surgery and exercise training.

Obesity is associated with an abnormal gene expression profile^49^ and skeletal muscle phenotype.^50^ Furthermore, bariatric surgery can further aggravate muscle atrophy and strength loss. Within this scenario, we examined the extent to which exercise may rescue muscle genotype and phenotype in patients who underwent bariatric surgery comparing the experimental groups (i.e. RYGB + ET) and RYGB groups (at POST9) with a healthy lean group of reference. RYGB + ET showed a much lower number of differentially expressed genes when compared with healthy lean individuals than did RYGB (276 vs. 544 genes, respectively), and similar enrichment scores for all pathways analysed in the current study. In contrast, RYGB showed higher enrichment scores in the ubiquitin‐mediated proteolysis and FOXO signalling pathway which may negatively influence the chronic skeletal muscle adaptations. In agreement with the aforementioned results, RYGB + ET showed comparable values of absolute and relative lower‐limb muscle strength and absolute upper‐limb muscle strength with the healthy control group, while RYGB exhibited reduced values of absolute and relative lower‐limb muscle strength and absolute upper‐limb muscle strength. Taken together, our findings indicate that exercise following bariatric surgery can shift the skeletal muscle gene expression profile closer to what is expected for healthy lean individuals and, mostly important, that exercise training is effective in fully (e.g. muscle strength and muscle fibre capillarization) or partially (e.g. functionality) recovering some muscle phenotypical aspects that are negatively impacted by bariatric surgery. Nevertheless, caution should be exercised in the interpretations of these results, as our healthy lean control group was limited in size, which may be underpowered to detect statistically significant differences.

The results of this study are not free of limitations. Our findings are confined to the main characteristics of the participants (i.e. middle‐aged women, with severe obesity, with a generally lower level of education, and economic status and limited by the duration of the follow‐up period.

Despite excellent reliability scores, strength and functionality tests were performed by an unblinded investigator. Histological (i.e. cross‐sectional area of muscle fibres, satellite cells, and capillarization) and molecular (i.e. RNA‐seq, protein, and gene expression) analyses were performed in a sub‐sample of patients, and the experimental groups (RYGB and RYGB + ET at POST9) were compared with small sample of healthy lean control group, which may be underpowered for some measures.

In conclusion, a 6 month, supervised training programme was an effective strategy to mitigate the commonly observed fat‐free mass loss and to reverse muscle atrophy and strength loss among women who had bariatric surgery, which may be partially explained by increases in capillarization, satellite cell content, and suppressed muscle protein breakdown mediated by ubiquitin‐proteasome system. Of relevance, exercise resulted in a skeletal muscle genotype and phenotype that more closely resembles that of healthy lean individuals. Collectively, this study provides evidence—from gene to function—that strongly supports the incorporation of exercise into the post‐operative routine of bariatric patients to counteract the adverse effects of surgery on muscle mass and function.

## Trial registration

Clinicaltrial.gov: NCT02441361.

## Conflict of interest

The authors have declared that no conflict of interest exists.

## Funding

The authors acknowledge the support by São Paulo Research Foundation (FAPESP—2016/10993‐5), the Brazilian National Council for Scientific and Technological Development (CNPq—grant 400157/2016‐0 and 301571/2017‐1). The study is also partially supported by NIGMS U54GM104940, and NIGMS P20GM103528 from NIH, USA, and by the National Medical Research Council and Ministry of Health, Singapore (WBS R913200076263). S.G., H.R. and B.G. are supported by grants from the Conselho Nacional de Pesquisa e Desenvolvimento (CNPq, 166622/202‐6; 488242/2018‐9; and 301914/2017‐6) B.G. and S.G. is also supported by a grant the Sao Paulo Research Foundation (FAPESP, 2017/13551‐2; 2020/08091‐9).

## Supporting information


**Table S1.** List of reagents and resources.
**Table S2.** Dietary intake.
**Figure S1.** Study Recruitment. RYGB+ET: Roux‐en‐Y Gastric Bypass plus Exercise Training group; RYGB: Roux‐en‐Y Gastric Bypass plus non‐exercise. ITT: intention‐to‐treat.
**Figure S2.** Expression of Genes and Protein Related to Angiogenesis. RYGB+ET (*n* = 14): Roux‐en‐Y Gastric Bypass plus Exercise Training group; RYGB (n = 14): Roux‐en‐Y Gastric Bypass plus non‐exercise. Gene expression of the *HIF1‐α*, *ANGPT1*, *ANGPT2*, *MDM2*, *TEK* and *THSP1* (Panel A, B, C, D, E and F, respectively). Protein expression of the *VEGF* (Panel G). Representative image of the Western blot bands for *VEGF* (Panel H). Data are expressed as mean ± SD. PRE: before surgery (baseline); POST3: 3 months following surgery; POST9: 9 months following surgery. ^ indicates *P* < 0.05 for main effect of time; * indicates P < 0.05 in comparison to PRE; # indicates P < 0.05 in comparison to POST3; $ indicates P < 0.05 for between‐group comparison at POST9.
**Figure S3.** Expression of Genes and Proteins Related to Protein Breakdown mediated by Autophagic System. RYGB+ET (*n* = 14[8 for western blot]): Roux‐en‐Y Gastric Bypass plus Exercise Training group; RYGB (n = 14[8 for western blot]): Roux‐en‐Y Gastric Bypass plus non‐exercise. Gene expression of the *BNIP3*, *BECN1* and *CTSL1* (Panel A, B and C, respectively). Protein expression of the *Beclin‐1* and *LC‐3* (Panel D and E, respectively), Representative image of the Western blot bands for *Beclin‐1* and *LC‐3* (Panel H). Data are expressed as mean ± SD. PRE: before surgery (baseline); POST3: 3 months following surgery; POST9: 9 months following surgery. ^ indicates *P* < 0.05 for main effect of time.
**Figure S4**. Expression of Proteins Related to mTOR Pathway Along the Intervention Period. RYGB+ET (*n* = 12): Roux‐en‐Y Gastric Bypass plus Exercise Training group; RYGB (n = 12): Roux‐en‐Y Gastric Bypass plus non‐exercise. Protein expression of the *p‐mTOR*, *mTOR*, *p‐mTOR/mTOR* ratio (Panel A, B and C, respectively). Protein expression of the *p‐p70S6K*, *p70S6K*, *p‐p70S6K/p70S6K* ratio (Panel D, E and F, respectively). Protein expression of the *p‐4E‐BP1*, *4E‐BP1*, *p‐4E‐BP1/4E‐BP1* ratio (Panel G, H and I, respectively). Representative image of the Western blot of the proteins related to *mTOR* pathway (Panel J). Data are expressed as mean ± SD. PRE: before surgery (baseline); POST3: 3 months following surgery; POST9: 9 months following surgery. ^ indicates *P* < 0.05 for main effect of time.
