# Supplementary material for: A randomized clinical trial on the effects of exercise on muscle remodelling following bariatric surgery
Source: J Cachexia Sarcopenia Muscle. 2021 Oct 19;12(6):1440–55. doi: 10.1002/jcsm.12815 (PMC8718087; doi:10.1002/jcsm.12815)
Supplement: Supplementary file 1 — Table S1. List of reagents and resources. Table S2. Dietary intake. Figure S1. Study Recruitment. RYGB+ET: Roux‐en‐Y Gastric Bypass plus Exercise Training group; RYGB: Roux‐en‐Y Gastric Bypass plus non‐exercise. ITT: intention‐to‐treat. Figure S2. Expression of Genes and Protein Related to Angiogenesis. RYGB+ET (n = 14): Roux‐en‐Y Gastric Bypass plus Exercise Training group; RYGB (n = 14): Roux‐en‐Y Gastric Bypass plus non‐exercise. Gene expression of the HIF1‐α, ANGPT1, ANGPT2, MDM2, TEK and THSP1 (Panel A, B, C, D, E and F, respectively). Protein expression of the VEGF (Panel G). Representative image of the Western blot bands for VEGF (Panel H). Data are expressed as mean ± SD. PRE: before surgery (baseline); POST3: 3 months following surgery; POST9: 9 months following surgery. ^ indicates P < 0.05 for main effect of time; * indicates P < 0.05 in comparison to PRE; # indicates P < 0.05 in comparison to POST3; $ indicates P < 0.05 for between‐group comparison at POST9. Figure S3. Expression of Genes and Proteins Related to Protein Breakdown mediated by Autophagic System. RYGB+ET (n = 14[8 for western blot]): Roux‐en‐Y Gastric Bypass plus Exercise Training group; RYGB (n = 14[8 for western blot]): Roux‐en‐Y Gastric Bypass plus non‐exercise. Gene expression of the BNIP3, BECN1 and CTSL1 (Panel A, B and C, respectively). Protein expression of the Beclin‐1 and LC‐3 (Panel D and E, respectively), Representative image of the Western blot bands for Beclin‐1 and LC‐3 (Panel H). Data are expressed as mean ± SD. PRE: before surgery (baseline); POST3: 3 months following surgery; POST9: 9 months following surgery. ^ indicates P < 0.05 for main effect of time. Figure S4. Expression of Proteins Related to mTOR Pathway Along the Intervention Period. RYGB+ET (n = 12): Roux‐en‐Y Gastric Bypass plus Exercise Training group; RYGB (n = 12): Roux‐en‐Y Gastric Bypass plus non‐exercise. Protein expression of the p‐mTOR, mTOR, p‐mTOR/mTOR ratio (Panel A, B and C, respectively). Protein ex [file JCSM-12-1440-s001.docx]

SUPPLEMENTARY TABLES

| **Table S1. List of reagents and resources.** |  |  |
| --- | --- | --- |
| **REAGENT or RESOURCE** | **SOURCE** | **IDENTIFIER** |
| ***Commercial assays*** |  |  |
| RNeasy Fibrous Tissue Mini kit | Qiagen | Cat# 74704 |
| SuperScript™ III Platunum ™ One-Step qRT-PCR kit | ThermoFisher Scientific | Cat# 11732020 |
| QuantSeq 3’mRNA-Seq Library Prep Kit FWD for Illumina | Lexogen | Cat# 015.96 |
| Pierce™ BCA Protein Assay | ThermoFisher Scientific | Cat# 23225 |
| ***Antibodies*** |  |  |
| Anti-Laminin | Abcam (1:100) | Cat# ab11575 |
| Anti-MHCI | DSHB (1:75) | Cat# A4.951 |
| Anti-Pax7 | DSHB (neat) | Cat# Pax7 |
| Anti-CD31 | Abcam (1:20) | Cat# ab28364 |
| Goat anti-rabbit IgG (H+L), Alexa Fluor 488 | ThermoFisher Scientific (1:200) | Cat# A-32731 |
| Goat anti-rabbit IgG (H+L), Alexa Fluor 568 | ThermoFisher Scientific (1:200) | Cat# A-11036 |
| Goat anti-mouse IgG (H+L), Alexa Fluor 594 | ThermoFisher Scientific (1:1000) | Cat# A-11032 |
| p-mTOR | Cell Signaling (1:1000) | Cat# 5536 |
| mTOR | Cell Signaling (1:1000) | Cat# 2983 |
| p-p70 S6K | Cell Signaling (1:1000) | Cat# 9204 |
| p70 S6K | Cell Signaling (1:1000) | Cat# 2708 |
| p-4E-BP1 | Cell Signaling (1:1000) | Cat# 2855 |
| 4E-BP1 | Cell Signaling (1:1000) | Cat# 9644 |
| LC3A/B | Cell Signaling (1:1000) | Cat# 12741 |
| Beclin-1 | Cell Signaling (1:1000) | Cat# 3495 |
| MuRF-1 | Santa Cruz (1:1000) | Cat# 33782 |
| Atrogin-1 | Santa Cruz (1:200) | Cat# 134397 |
| VEGF | Cell Signaling (1:1000) | Cat# 2463 |
| ***Chemicals and Recombinant Proteins*** |  |  |
| TRIzol Reagent | Invitrogen | Cat# 15596018 |
| Chloroform | Sigma Aldrich | Cat# C7559-5VL |
| Ethanol absolute ≥ 99.8% (GC) | Sigma Aldrich | Cat# 24102-2.5L-R |
| DEPC water | ThermoFisher Scientific | Cat# 750023 |
| Sybr Green MasterMix | ThermoFisher Scientific | Cat# K0222 |
| ROX Reference Dye | ThermoFisher Scientific | Cat# 12223012 |
| TBS | Sigma Aldrich | Cat# T5912-1L |
| PBS | Sigma Aldrich | N/A |
| Goat serum | Sigma Aldrich | Cat# G9023-10ML |
| Triton X-100 | Sigma Aldrich | Cat# T8787-250mL |
| 2-Mercaptoethanol | Sigma Aldrich | Cat# M3148 |
| PMSF | Sigma Aldrich | Cat# 93482 |
| RIPA buffer | ThermoFisher Scientific | Cat# 89900 |
| Halt™ Protease and Phosphatase Inhibitor Cocktail (100X) | ThermoFisher Scientific | Cat# 78444 |
| Novex™ 4-20% Tris-Glycine Mini Gel, 15 well | ThermoFisher Scientific | Cat# XP04205BOX |
| PVDF | BIO-RAD | Cat# 1620177 |
| Protein ladder | GE Healthcare | Cat# RPN800E |
| Ponceau S staining | Sigma Aldrich | Cat# P7170-1L |
| BSA | Sigma Aldrich | Cat# A7906 |
| Tween-20 | Sigma Aldrich | Cat# P9416-100ML |
| SuperSignal West Femto Chemilumininescent Substrate | ThermoFisher Scientific | Cat# 34096 |
| HCl | Sigma Aldrich | Cat# H1758-500ML |
| ***Oligonucleotides*** |  |  |
| Human *MuRF-1* Forward  (TGAGCCAGAAGTTTGACACG);  Human *MuRF-1* Reverse  (GATGAGTTGCTTGGCAGTCA) | Invitrogen | N/A |
| Human *Atrogin-1* Forward  (TCACAGCTCACATCCCTGAG);  Human *Atrogin-1* Reverse  (GACTTGCCGACTCTTTGGAC) | Invitrogen | N/A |
| Human *BNIP3* Forward  (AAAATACTGCTGGACGCACA);  Human *BNIP3* Reverse  (GAATATTTTCCGGCCGACTT) | Invitrogen | N/A |
| Human *BECN1* Forward  (TCACCATCCAGGAACTCACA);  Human *BECN1* Reverse  (TTCAGTCTTCGGCTGAGGTT) | Invitrogen | N/A |
| Human *CTSL1* Forward  (GTGGACATCCCTAAGCAGGA);  Human *CTSL1* Reverse  (CATTCTTCACCCCAGCTGTT) | Invitrogen |  |
| Human *HIF*-*1α* Forward  (CCCAATGGATGATGACTTCC);  Human *HIF*-*1α* Reverse  (TGGGTAGGAGATGGAGATGC) | Invitrogen | N/A |
| Human *ANGPT1* Forward  (GGGGGAGGTTGGACTGTAAT);  Human *ANGPT1* Reverse  (GAATAGGCTCGGTTCCCTTC) | Invitrogen | N/A |
| Human *ANGPT2* Forward  (TGCAAGTGCTGGAGAACATC);  Human *ANGPT2* Reverse  (GTTAACTTCCGCGTTTGCTC) | Invitrogen | N/A |
| Human *MDM2* Forward  (GATTCCAGCTTCGGAACAAG);  Human *MDM2* Reverse  (CCTGATCCAACCAATCACCT) | Invitrogen | N/A |
| Human *TEK* Forward  (TACACCTGCCTCATGCTCAG);  Human *TEK* Reverse  (TGTGCAGTTCACAAGCCTTC) | Invitrogen | N/A |
| Human *THBS1* Forward  (CCTCAATGAACGGGACAACT);  Human *THBS1* Reverse  (GTTCTGGTGGCCATCTTCAT) | Invitrogen | N/A |
| Human *β2M* Forward  (GGTTTACTCACGTCATCCAGC)  Human *β2M* Reverse  (ACACGGCAGGCATACTCAT) | Invitrogen | N/A |
| ***Software*** |  |  |
| GraphPad PRISM 7 | 1992-2017 GraphPad Prism Software | N/A |
| SAS 9.2 | SAS | N/A |
| Image J | NIH | *https://imagej.nih.gov/ij/* |
| CoreScan™ software | GE Healthcare | Cat# 2016v17 |
| Computer-generated randomization | Website | *www.randomization.com* |
| ***Other*** |  |  |
| Polar^®^ heart rate monitor | Polar | Cat# S810i |
| Lunar iDXA^®^ | GE Healthcare | N/A |
| Indirect calorimetry system | Cortex | Cat# Metalyzer IIIB^®^ |
| Treadmill | Micromed | Cat# Centurion 200^®^ |
| Eletrocardiogram | Micromed | Cat# Ergo PC Elite^®^ |
| Nikon Eclipse 90i microscope | Nikon | Cat# M319E |
| Polytron™ PT 1300 | Kinematica | Cat# 08-451-71 |
| NanoVue™ Plus Spectrophotometer | GE Healthcare | N/A |
| HiSeq 2500 | Illumina | N/A |
| Step One™ Real-Time PCR System | Applied Biosystems | Cat# 4376357 |
| Mini Gel Tank | ThermoFisher Scientific | Cat# A25977 |
| PowerPac Universal Power Supply | BIO-RAD | Cat# 1645070 |
| C-DiGit® Blot Scanner | LI-COR | Cat# 3600 |

**
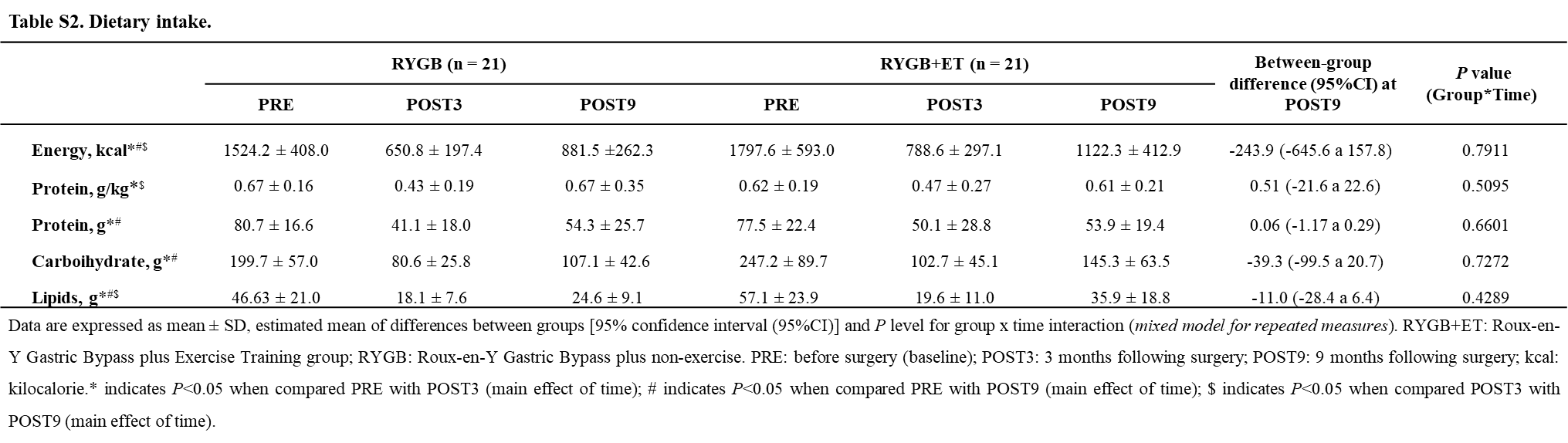
**

**SUPPLEMENTARY FIGURES**


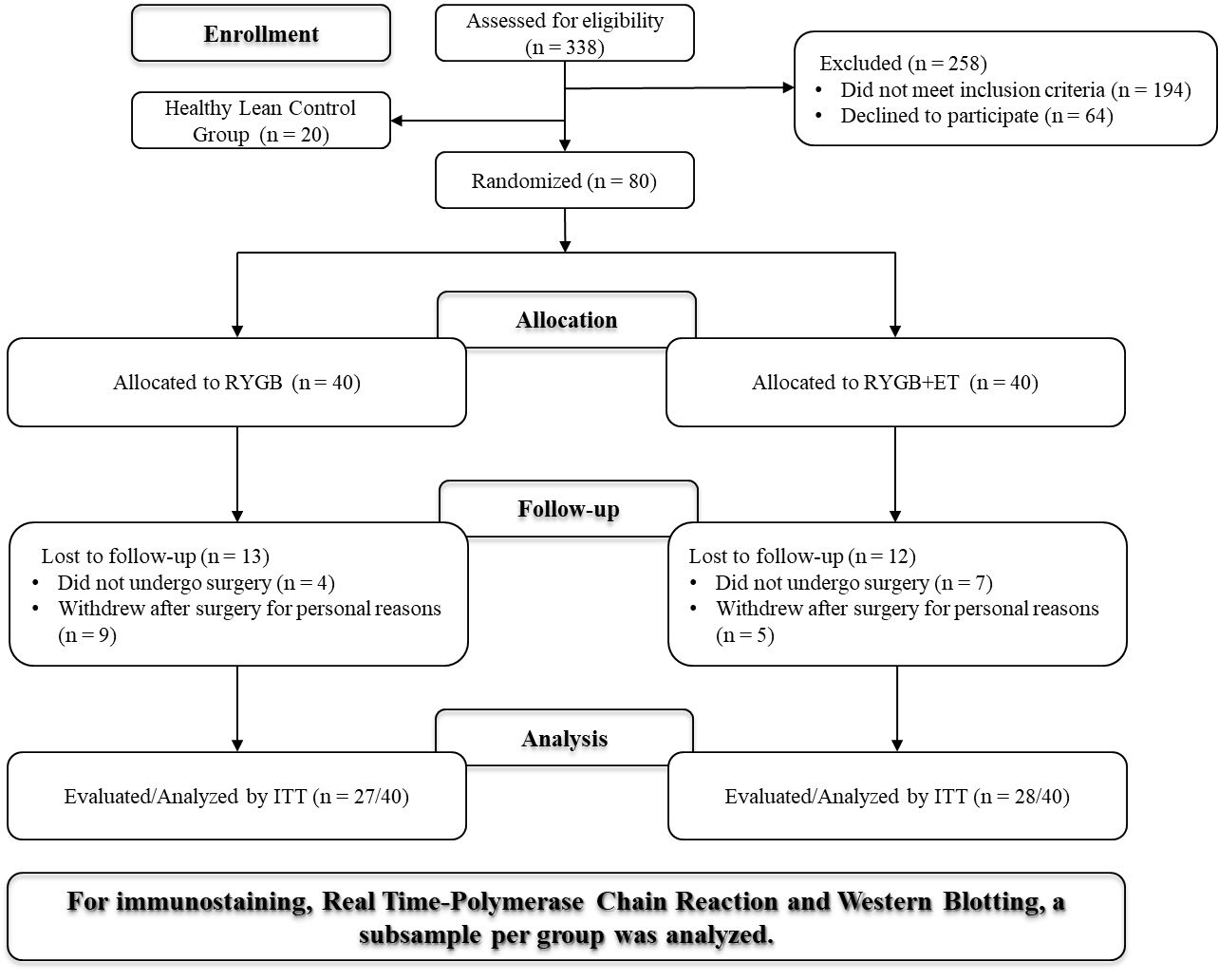
Figure S1. Study Recruitment. RYGB+ET: Roux-en-Y Gastric Bypass plus Exercise Training group; RYGB: Roux-en-Y Gastric Bypass plus non-exercise. ITT: intention-to-treat.

**Figure S2. Expression of Genes and Protein Related to Angiogenesis.** RYGB+ET (n = 14): Roux-en-Y Gastric Bypass plus Exercise Training group; RYGB (n = 14): Roux-en-Y Gastric Bypass plus non-exercise. Gene expression of the *HIF1-α*, *ANGPT1*, *ANGPT2*, *MDM2*, *TEK* and *THSP1* (Panel A, B, C, D, E and F, respectively). Protein expression of the *VEGF* (Panel G). Representative image of the Western blot bands for *VEGF* (Panel H). Data are expressed as mean ± SD. PRE: before surgery (baseline); POST3: 3 months following surgery; POST9: 9 months following surgery. ^ indicates P<0.05 for main effect of time; * indicates P<0.05 in comparison to PRE; # indicates P<0.05 in comparison to POST3; $ indicates P<0.05 for between-group comparison at POST9.

**Figure S3. Expression of Genes and Proteins Related to Protein Breakdown mediated by Autophagic System.** RYGB+ET (n = 14[8 for western blot]): Roux-en-Y Gastric Bypass plus Exercise Training group; RYGB (n = 14[8 for western blot]): Roux-en-Y Gastric Bypass plus non-exercise. Gene expression of the *BNIP3*, *BECN1* and *CTSL1* (Panel A, B and C, respectively). Protein expression of the *Beclin-1* and *LC-3* (Panel D and E, respectively), Representative image of the Western blot bands for *Beclin-1* and *LC-3* (Panel H). Data are expressed as mean ± SD. PRE: before surgery (baseline); POST3: 3 months following surgery; POST9: 9 months following surgery. ^ indicates *P*<0.05 for main effect of time.

**
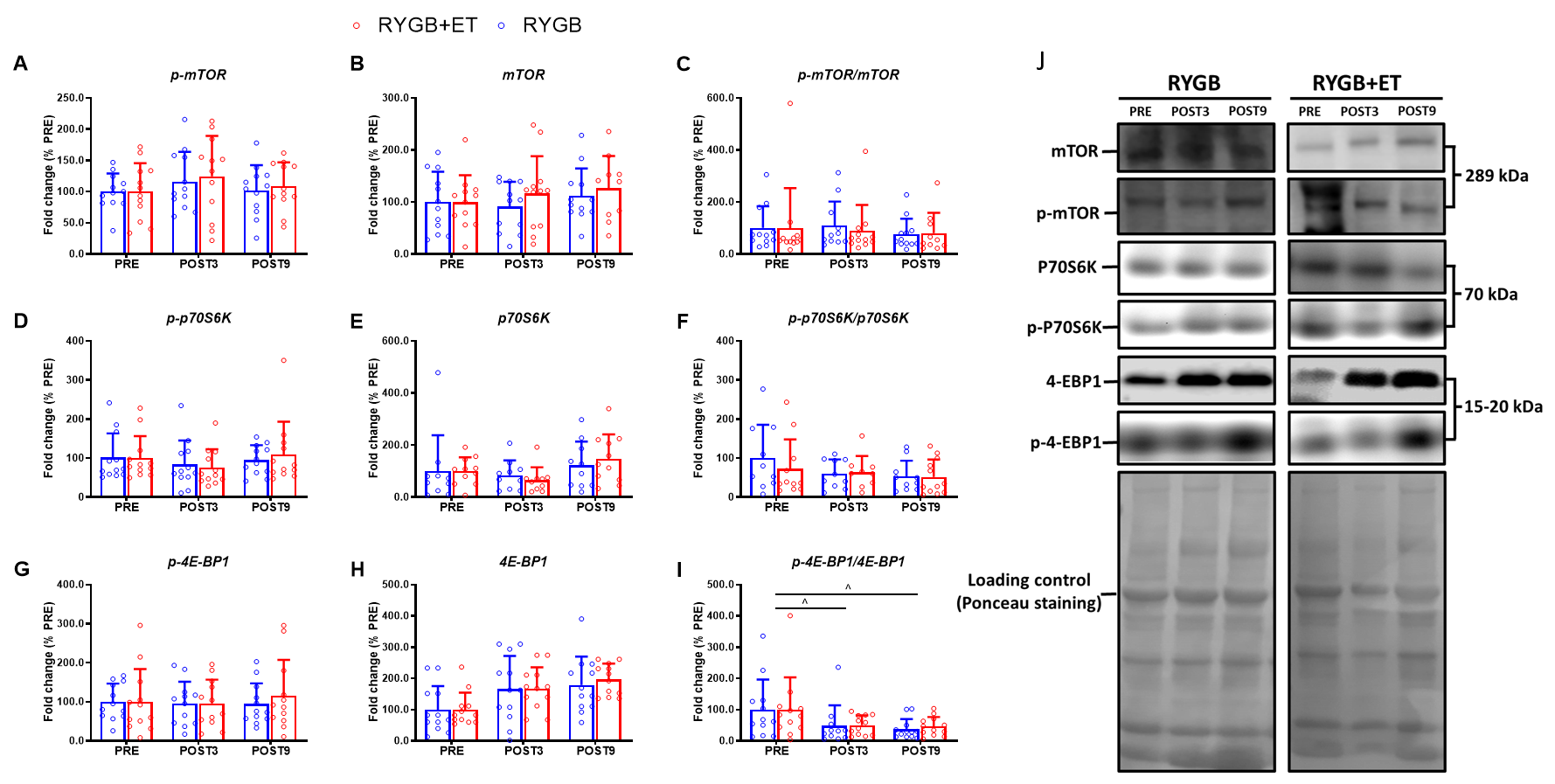
Figure S4. Expression of Proteins Related to mTOR Pathway Along the Intervention Period.** RYGB+ET (n = 12): Roux-en-Y Gastric Bypass plus Exercise Training group; RYGB (n = 12): Roux-en-Y Gastric Bypass plus non-exercise. Protein expression of the *p-mTOR*, *mTOR*, *p-mTOR/mTOR* ratio (Panel A, B and C, respectively). Protein expression of the *p-p70S6K*, *p70S6K*, *p-p70S6K/p70S6K* ratio (Panel D, E and F, respectively). Protein expression of the *p-4E-BP1*, *4E-BP1*, *p-4E-BP1/4E-BP1* ratio (Panel G, H and I, respectively). Representative image of the Western blot of the proteins related to *mTOR* pathway (Panel J). Data are expressed as mean ± SD. PRE: before surgery (baseline); POST3: 3 months following surgery; POST9: 9 months following surgery. ^ indicates *P*<0.05 for main effect of time.
